# ChatGPT (GPT-3.5) as an assistant tool in microbial pathogenesis studies in Sweden: a cross-sectional comparative study

**DOI:** 10.3352/jeehp.2023.20.32

**Published:** 2023-11-22

**Authors:** Catharina Hultgren, Annica Lindkvist, Volkan Özenci, Sophie Curbo

**Affiliations:** 1 Division of Clinical Microbiology, Department of Laboratory Medicine, ANA Futura, Karolinska Institutet, Huddinge, Sweden; 2 Division of Clinical Immunology, Department of Laboratory Medicine, ANA Futura, Karolinska Institutet, Huddinge, Sweden; Hallym University, Korea

**Keywords:** Artificial intelligence, Dental students, Educational personnel, Microbiology, Sweden

## Abstract

ChatGPT (GPT-3.5) has entered higher education and there is a need to determine how to use it effectively. This descriptive study compared the ability of GPT-3.5 and teachers to answer questions from dental students and construct detailed intended learning outcomes. When analyzed according to a Likert scale, we found that GPT-3.5 answered the questions from dental students in a similar or even more elaborate way compared to the answers that had previously been provided by a teacher. GPT-3.5 was also asked to construct detailed intended learning outcomes for a course in microbial pathogenesis, and when these were analyzed according to a Likert scale they were, to a large degree, found irrelevant. Since students are using GPT-3.5, it is important that instructors learn how to make the best use of it both to be able to advise students and to benefit from its potential.

## Graphical abstract

[Fig f3-jeehp-20-32]

## Background/rationale

ChatGPT (Chat Generative Pre-trained Transformer) is a freely accessible artificial intelligence (AI) chatbot model that was developed on the concept of reinforcement learning from human feedback [1]. It can generate human-like conversations and has, for example, been used to aid in writing, although it is not considered advanced enough to fully function as a writer of scientific literature [2,3]. When GPT-3.5 (https://chat.openai.com/) was publicly released on November 30, 2022, it spread rapidly among students. In faculties of education, this raised concerns regarding academic dishonesty and that students would eventually lose their ability to produce original ideas, develop critical thinking, and present proper arguments to prove a point [4]. Studies have also shown that GPT-3.5 performs at or near the passing threshold on the United States Medical Licensing Exam [5], suggesting not only that GPT-3.5, and with time more advanced chatbots, may have an impact in clinical medicine at large, but also that we need to rethink how to assess learning. In a subsequent study, GPT-3.5 was found to be preferred both in terms of quality and empathy over physician responses in a social media forum [6]. Despite the many limitations of GPT-3.5 and challenges in implementing AI tools in the medical field, these tools are advancing further, and we are currently learning how to best use AI tools in the field of higher education. GPT-3.5 has been used to assist in curriculum design and the development of assessment strategies and teaching methods, as well as serving as a virtual teaching assistant [7]. Here, we further evaluate the usefulness of GPT-3.5 to assist in the development of detailed learning outcomes and to answer student questions in a course on microbial pathogenesis for dental students at Karolinska Institutet.

## Objectives

This study aimed to compare the knowledge and interpretation ability of GPT-3.5 with those of teachers at Karolinska Institutet in a course on microbial pathogenesis. To evaluate both whether teachers could implement GPT-3.5 to answer students’ questions related to the course content and how teachers could make use of GPT-3.5 when designing detailed intended learning outcomes, the following was investigated: the ability of GPT-3.5 to interpret questions from students and give concise and correct facts and the resemblance to a teacher’s replies to the same questions, and the usefulness of GPT-3.5 in creating detailed intended learning outcomes in the same course.

## Ethics statement

Ethical approval was not required for this study, as per the Swedish Ethical Review Authority tool. This study did not include a clinical trial and did not collect any personal data. It was exempted from the requirement to obtain informed consent.

## Study design

This cross-sectional comparison study was conducted to compare the ability of GPT-3.5 and teachers to answer questions from dental students and construct detailed intended learning outcomes.

## Setting

**Fig. 1.**
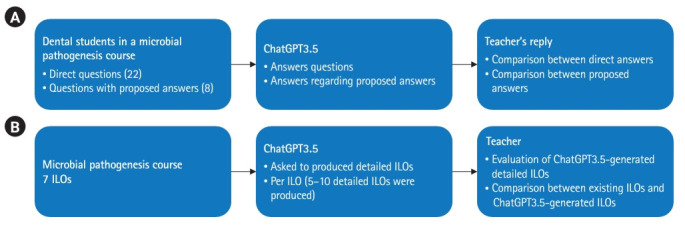
Outline of the study. (A) Questions from students and (B) intended learning outcomes (ILOs).

The questions from the students and replies from the teachers were obtained from an online discussion forum during a course in microbial pathogenesis for dental students during their third semester at Karolinska Institutet, Sweden in September 2022 (Dataset 1). The teachers were at the time unaware that their answers would be used in the current study. The same questions were administered to GPT-3.5 in May 2023 (Dataset 1). The 7 different intended learning outcomes were individually supplied to GPT-3.5, with a prompt to create new detailed intended learning outcomes for each individual intended learning outcome (Dataset 2). The intended learning outcomes created by GPT-3.5 were subsequently compared to the detailed intended learning outcomes created for the course in 2022 by teachers (Fig. 1).

## Participants

The questions were asked by 22 dental students who took the course on microbial pathogenesis in September 2022. The questions were mainly of the second-order category. One teacher replied to all questions initially, and 2 other teachers retrospectively reviewed the initial teacher’s responses during March/April 2023. There were 7 intended learning outcomes in the microbial pathogenesis course. There were no exclusion criteria; thus, all questions and intended learning outcomes were included. The same questions and intended learning outcomes were asked to GPT-3.5 in May 2023.

## Variables

The items’ knowledge level and resemblance to the teacher’s answers were the variables.

## Data sources/measurement

The teacher’s answers to students’ questions about basic and oral microbiology and immunology were compared with the responses of GPT-3.5. The correct answer rate of GPT-3.5 was evaluated according to a Likert scale between 1 to 5; where 1 indicates an irrelevant response or a situation where the question was not understood and 5 corresponds to a correct answer with additional information. The answers from GPT-3.5 were also compared to teacher’s answers according to a different Likert scale, where 1 indicates an incorrect answer and 5 denotes an extended answer and suggests further reading compared to the teacher’s answer. The authors also evaluated the resemblance of detailed intended learning outcomes generated by GPT-3.5 to those generated by teachers according to a Likert scale, where 1 indicates irrelevant or misleading intended learning outcomes, and 5 corresponds to relevant, extended, and new intended learning outcomes.

## Bias

No notifiable bias could be detected because all questions addressed by the students and all detailed intended learning outcomes were included in the study.

## Study size

A sample size calculation was not required since all questions and intended learning outcomes were included.

## Statistical methods

Descriptive statistics were used to analyze GPT-3.5’s scores for correctness and comparisons to the teacher’s replies using Excel ver. 2016 (Microsoft Corp.).

## Main results

### The ability of GPT-3.5 to answer students’ questions

All 22 questions from the students generated a relevant response by GPT-3.5 (Table 1, Dataset 1). When reviewing the replies by GPT-3.5 in detail, it was evident that the replies were both relevant and correct (mean value=4.4). In the majority of the replies of GPT-3.5 the answers were extended, suggesting further reading (16/22 or 72.7%) and, in comparison to the answers given by the teacher, it was evident that GPT-3.5 both elaborated on most questions and gave longer replies than the teacher. GPT-3.5 was also perceived as more polite than the teacher (Dataset 1).

### The usefulness of GPT-3.5 in constructing detailed learning outcomes

The 7 different intended learning outcomes were individually input into GPT-3.5 with the request to produce detailed intended learning outcomes for each intended learning outcome. Thereafter, the detailed intended learning outcomes were compared with the detailed intended learning outcomes used in the course curriculum during September 2022. Overall, except for one intended learning outcome, the detailed intended learning outcomes constructed by GPT-3.5 were very extensive, too advanced, and in some cases even misleading (Dataset 2). In one case, the intended learning outcome “Reflect on the importance of different dental biomaterials used in dentistry and their impact on oral health and the environment” was completely misunderstood by GPT-3.5, which created few and partially incorrect detailed intended learning outcomes (Dataset 2).

## Key results

The aim of this study was to compare the knowledge and interpretation ability of GPT-3.5 with those of teachers at Karolinska Institutet in a course on microbial pathogenesis for dental students. We found that GPT-3.5 had the ability to interpret questions from students and give concise and correct facts in response, and in most of the replies (73%), the answers were longer than the teacher’s reply. Furthermore, GPT-3.5 was able to construct detailed intended learning outcomes although these were very extensive and, in some cases, even misleading.

## Interpretation

**Fig. 2.**
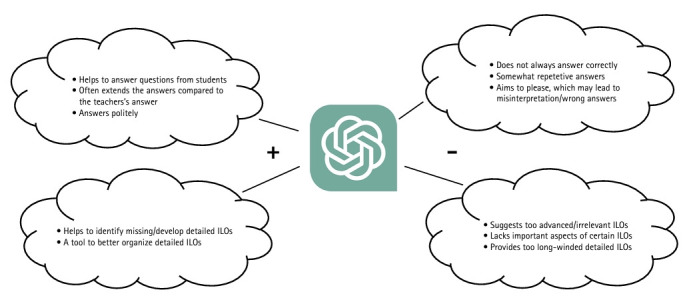
Pros and cons of using GPT-3.5 as an aid in higher education. ILOs, intended learning outcomes.

GPT-3.5 was able to understand the students’ questions and in many cases, both gave longer and more extended answers than the teacher. GPT-3.5 is instructed to be pleasant and reply more politely, which may lead to misinterpretation/wrong answers (Fig. 2). We believe that one reason why the teacher’s answers sometimes were short and concise was to engage the student more actively through finding answers on his/her own. Another reason could be that the teacher wanted to stress a specific part of an answer that was particularly important. GPT-3.5 was a useful tool to better organize detailed intended learning outcomes and provided help in identifying missing learning outcomes or developing detailed intended learning outcomes. However, some major drawbacks were that the suggested detailed intended learning outcomes often were too advanced or irrelevant and long-winded. Important aspects of certain intended learning outcomes were also missing (Fig. 2). Caution must be used when interpreting the data given the limited number of questions and intended learning outcomes analyzed.

## Comparison with previous studies

In accordance with previous studies, we found that GPT-3.5 was more polite and also provided longer answers than the teacher [6]. The assistance of GPT-3.5 in the construction of detailed intended learning outcomes was limited in agreement with a study by Lee et al., where GPT-3.5 was used for curriculum design [7].

## Limitations/generalizability

There are limitations in the study concerning the small number of questions that were used to evaluate ChatGPT3.5’s capacity to reply correctly and guide students in the field. Most questions concerned basic microbiology or immunology, which are well-known fields for which replies may be easier than for a narrower subject such as oral microbiology. This was evident when GPT-3.5 was used to create new detailed intended learning outcomes. Furthermore, the instructions given to GPT-3.5 might have been incomplete, which could have influenced the outcome. When given proper instructions, we believe that GPT-3.5 can be useful and important for other subjects, both in teaching and learning.

## Suggestions

GPT-3.5 is already used by many students; therefore, we believe that it is important for faculty members to learn about the advantages and disadvantages of GPT-3.5, so that they can advise students on how to use GPT-3.5 in a relevant fashion. Since we experienced that GPT-3.5 is instructed to be pleasant, the way questions are phrased is important and may therefore be a potential problem. One way to address this would be to give more detailed information on what is wanted (e.g., limiting the detailed outcomes to dentistry).

## Conclusion

GPT-3.5’s knowledge, interpretation, and ability to answer students’ questions in microbiology were found to be comparable to those of a teacher. However, GPT-3.5 is hampered by its instructions to be pleasant to the reader and it requires knowledge to really know if a given answer is correct. GPT-3.5 lacks knowledge in constructing detailed intended learning outcomes, but has the potential to become a useful tool to assist in teaching and education in general.

## Figures and Tables

**Figure f3-jeehp-20-32:**
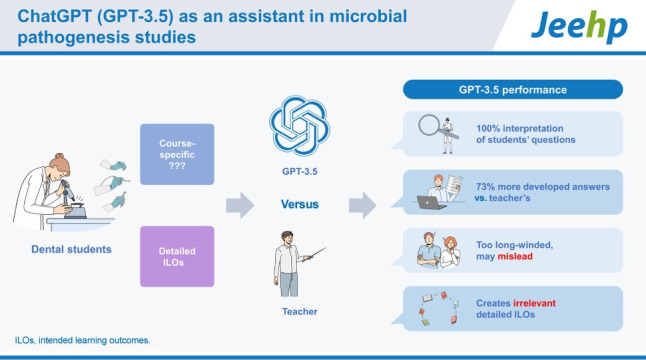


**Table 1.** Nature of replies from GPT-3.5 to students’ questions in terms of percentage agreement (numbers in parenthesis) and comparison with the teacher’s reply according to a Likert scale

Nature of reply from GPT-3.5	Percentage agreement or Likert scale rating
Interpretation of question	100.0% (22/22)
Extends the answer and suggests further reading	72.7% (16/22)
Gives concise and correct facts (Likert 1–5)	4.4
Comparison with teacher’s reply (Likert 1–5)	3.9

Likert scale where 1 corresponds to an incorrect or irrelevant answer and 5 indicates a correct answer with additional relevant information.

## Footnotes

**Data availability**
Data files are available from Harvard Dataverse: https://doi.org/10.7910/DVN/KGN6J3 Dataset 1. Students’ questions and GPT-3.5’s answers versus the teacher’s answers.

Dataset 2. Intended learning outcomes and detailed contents.

## Supplementary materials

Supplement 1. Audio recording of the abstract.
